# Mobile health technology in providing maternal health services – Awareness and challenges faced by pregnant women in upper West region of Ghana

**DOI:** 10.1016/j.puhip.2023.100407

**Published:** 2023-06-29

**Authors:** E. Bekyieriya, S. Isang, B. Baguune

**Affiliations:** a REJ Institute, Research and ICT Consultancy Services, P.O. Box TL1139, Tamale, Ghana; b School of Hygiene, Environmental Health Programme, Ministry of Health, Tamale, Ghana; c Ghana School of Law, Kwame Nkrumah University Science Technology, Kumasi, Ghana

**Keywords:** Mobile health technology, Maternal health services, Challenges, Pregnant women, Upper west region, Ghana

## Abstract

**Objectives:**

The study assessed awareness on Mobile Health (mhealth) Technology as well as challenges pregnant women encounter in the utilization of mhealth technology to improve maternal health in rural settings in the Upper West Region (UWR) of Ghana.

**Study design:**

The study was an exploratory design that employed the qualitative method of data collection.

**Methods:**

Semi-structured interview guide was used to conduct six (6) Focus Group Discussions (FGDs) and nine (9) Key Informant Interviews (KIIs) among pregnant women and health workers respectively from three (3) selected rural districts in the Upper West Region. Data was collected in August 2020. Thematic analysis was conducted and some statements from participants were presented verbatim to illustrate the themes realized.

**Results:**

Participants were aware of the mhealth intervention that had been implemented by Savanna Signatures in their districts. Major sources of information on the mhealth services were from durbars, health education sessions and health care providers. Challenges faced by pregnant women, in the mhealth technology intervention were; financial challenges, lack of mobile network connectivity, lack of electricity in some rural areas, low female literacy rate at household level and cultural barriers.

**Conclusion:**

The Savanna Signatures mhealth intervention is widely known but some challenges exist that impede the smooth implementation of the intervention. The mhealth technology intervention implementers should partner with other sectors and policy makers to address the challenges identified by the study.

## 1 Introduction

Science, Technology, and Innovation have been identified as a fulcrum way of achieving the Sustainable Developing Goals (SDGs) in the 2030 Agenda. In Ghana, like in many other Sub-Saharan African (SSA) nations, maternal mortality remains a severe issue among women of reproductive age (15–49 years), with 1 in 16 women dying as a result of pregnancy and delivery [[1], [2], [3]]. In the sector of women's health, mhealth technologies are playing a critical role in achieving SDG 3 target 1: “saving the lives of women in some of the most vulnerable communities”. Over 99% of maternal deaths among poor and rural women in developing nations are preventable [4]. Rural women in SAA had a far higher maternal mortality rate (MMR) (640 per 100,000 live births) than their urban counterparts (447 deaths per 100,000 live births) [5]. MMR in Ghana is over 380 per 100,000 live births [4], which is much higher than global norms of 216 deaths per 100,000 live births [4]. Rural regions in most poor nations, such as Ghana, have less access to healthcare services than metropolitan areas. These problems are especially acute in Ghana's Upper West Region (UWR), where there are a scarcity of physicians, nurses, and other health-care personnel [6].

Posited by World Health Organization (WHO), the building of our health system hinges on six blocks that may be used to increase health-care access and coverage [7]. One of these six building blocks is the adoption and implementation of mhealth. Mhealth, is defined as “the use of mobile wireless technologies for public health” [7]. Mhealth refers to all technologically based platforms that are used to improve patient care, monitoring, and health service delivery remotely. Mhealth serves to enhance the efficiency of healthcare delivery and provide chances to improve the quality and quantity of life [8]. WHO recognizes scientific applications as one of the health care pillars for creating pathways and the delivery of quality healthcare services, with universal mhealth being identified as a potential healthcare tool because of its widespread adoption and use by people, including healthcare professionals [7]. Notwithstanding this, the Ghanaian public healthcare system still struggles with access and quality of care. High maternal mortality and neonatal deaths are still posing serious concern in the country's public health issues, particularly in the rural districts [3].

Over the last decade, Information and Communication Technologies (ICTs) have grown significantly, resulting in socioeconomic development that is the cornerstone for mHealth [9]. In 2017, five billion people were connected to mobile services (including wireless networks), with an estimated 6 billion unique mobile subscribers by 2025 [10]. Mobile technologies include the iPad (Intelligent Pad), iPod (Intelligent Pod), cell phone, and personal digital assistant (PDA). Mobile phones have been used in a variety of health-care settings, including chronic disease treatment adherence, appointment reminders, public and primary health care, disease surveillance, epidemics, and telemedicine [11]. According to the third WHO Global Observatory on the state of mhealth among member countries, mhealth adoption is on the rise, with 114 countries using it to deliver a variety of healthcare services [7]. It is claimed that if citizens' mobile phones were equipped with basic healthcare information such as first aid, maternal and child health, and other topics, several lives could be saved [12]. Because of its widespread use, particularly in developing countries, the mobile phone has become an indispensable health device [12,13]. Mobile phones can provide information to the unreached, enable the activities of remote health workers, and reduce inefficiencies, all of which provide opportunities for development.

In Ghana, majority of the adult population owns more than one mobile phone [14]. This has provided a good opportunity for mhealth adoption in the country, however, pregnant women are still faced with challenges in assessing maternal health care services. Existing evidence shows that mHealth applications may be useful to improving maternal, neonatal and child health, including increased antenatal care attendance, facility usage and skilled attendance at birth, postnatal care attendance, and immunization rates [[15], [16], [17]]. Savanna Signatures is a Ghana-based registered non-governmental organization with a vision of a society where relevant information, knowledge and skills for development are enhanced by equal access to and use of Information and Communication Technology (ICT). In response to Ghana's high maternal mortality rate, Savanna Signatures piloted the Technology for Maternal and Child Health (T4MCH) program from 2017 to 2020. The T4MCH project was implemented in 33 health facilities in nine districts across the five regions of the northern part of Ghana, relies on using mobile phone technology to make maternal and child health (MCH) information easily accessible to expectant mothers and their families. The study was conducted in three rural districts in the Upper West Region of Ghana to ascertain the awareness of pregnant women on these mhealth services and the challenges they face in adopting the mhealth technology.

## 2 Methods

### 2.1 Study design

The study was an exploratory study. It employed the qualitative approach using KIIs and FGDs in the data collection process.

### 2.2 Study setting

The study was conducted in three T4MCH intervention pilot districts (Jirapa, Wa West and Wa East) in the UWR of Ghana, which is one of the poorest and the least developed regions of Ghana [13]. The intervention districts are among the hard-to-reach districts in the UWR in terms of access to health facilities, as a result of inadequate roads and poor infrastructure.

### 2.3 Population and sampling

The target population were women in fertility age (15–49 years). A woman who is pregnant and lives in the study area was the main criterion for inclusion. Random sampling was used to select the pregnant women. The sampling was done by using the registration numbers of the pregnant women at the health facilities at the Savana Signatures T4MCH intervention areas. Each pregnant woman selected through her registration number was contacted by a member of the research team to book appointment for the FGD. Purposive sampling was used to select midwives who were working in the Savana Signatures T4MCH intervention areas health facilities for the KIIs. In all, six FGDs (ranging from 8 to 10 participants per group) and nine KIIs were conducted for the study.

### 2.4 Data collection

Semi-structured interview guide was used to conduct Focus Group Discussions (FGDs) and Key Informant Interviews (KIIs) among study participants. The question items focused on awareness of pregnant women on the mhealth technology intervention in their districts and the challenges they face in utilizing the intervention activities. We conducted one-on-one in-depth interviews with 9 Midwives and 6 focus group discussion sessions. Overall, 52 pregnant women in groups of 8–10 participants per session took part in the FGDs. On the average, the in-depth interviews lasted for 45 min while the FGD sessions lasted for about 1 h, 30 min. The interviews were conducted in August 2020.

### 2.5 Analysis

All the audio recordings were transcribed while listening to the tapes and using the field notes taken. The transcripts were shared and read through thoroughly, independently, and repeatedly by the study team members to ensure completeness and accuracy of the transcriptions and also to obtain a sense of the data as a whole. Thematic analysis was carried out. Initial codes were produced by one of the authors from a list of ideas found to be interesting and relevant in the data which were later organized into meaningful groups. The generated codes were sorted out and merged to form potential themes. The initial themes were reviewed and refined into final themes taking into consideration internal homogeneity (ensuring everything in a theme is similar) and external heterogeneity (ensuring different contents in different themes). The themes were defined and named and detailed analysis conducted and written based on how they fit into the broader story of the data.

## 3 Results

Overall six FGDs involving a total of 52 pregnant women and KIIs involving 9 Midwives were conducted for the study (Table 1). The results are presented as themes based summaries and direct quotations from participants. The main themes were based on the objectives of the study; awareness of pregnant women on mhealth services and challenges they face in adopting the mhealth technology. Regarding knowledge, two sub themes were identified; awareness of mhealth technology in the district and source of information on awareness. For the challenges faced by pregnant women, five sub themes emerged from the main theme; financial challenges, lack of mobile network connectivity, lack of electricity in some rural areas, low female literacy rate at household level and cultural barriers.

**Table 1:** Type of Interview and number of Respondents per Intervention District.

District	KII	FGD	
		Number of FGDs	Number of participants
Jirapa	3	2	18
Wa West	3	2	17
Wa East	3	2	17
**Total**	**9**	**6**	**52**

### 3.1 Awareness of pregnant women on mhealth interventions

Participants were asked whether they were aware of the Savanna Signatures mhealth intervention in the district. The discussion showed that, participants were aware of the mhealth intervention that has been piloted by Savanna Signatures in their respective districts. The sources of information were from durbars, health education sessions and health providers. The following views are from participants on confirming their awareness and sources of information on the mhealth services by Savanna Signatures in their respective districts.

Views from pregnant women:

*“We are aware of the Savanna Signatures, …, when they came here first, they organized the whole community and informed us about their activities”*

*“Ooh, for Savanna Signatures, everybody here is aware of what they are doing …… …”*

*“The other time we were having a durbar on health promotion activities, they talked about some of the things Savanna Signatures is doing for the women including text messages and phone calls to check on pregnant women and also remind them about their Antenatal clinic appointments”.*

*“When I went for antenatal care, a midwife took my phone number and told me I will receive text messages and phone calls from her to ask me about my health”.*

A Midwife also said:

*“People in the communities are getting to know about the existence of Savanna Signatures through the durbars we normally hold to educate them. … once the woman visits the facility and is confirmed to be pregnant, the Midwife will take her through all the mhealth services available and coach her on how to access such services”.*

### 3.2 Challenges faced by pregnant women in the uptake of mhealth services

Participants were asked to elaborate challenges they face that are impediments in the uptake of the mhealth technology intervention. The discussions yielded five sub themes at the end of the saturation point. The sub themes are presented below in no order:

### 3.3 Financial challenges

There was a broader consensus that inability to procure and own mobile phones because of financial difficulties was one of the reasons why some pregnant women could not access services provided through the mhealth technology. A pregnant woman expressed her view as follows:

*“For me, I do not have mobile phone not because I don’t want it but because I cannot buy. How can I leave my children to sleep hunger while I use the money to buy phone? If I get I will use but I will not force myself”*

Another participant also said:

*“The mobile phones are costly that is why we cannot buy. You know we women don’t work for money, we work to assist our husbands feed the family so we do not have control over the household resources”.*

This is the view of one Midwife in support of the financial challenges faced by pregnant women:

*“… so, by introducing technology in maternal and child health, our biggest challenge has to do with the mothers' inability to afford the phones. You want them to go and buy phones, after buying the phone, now credit… so these are some of the challenges I think these mothers are likely to face”.*

Participants also consented that because of financial issues, males are normally the owners of mobile phones in the rural households making it difficult for women to have access to them. A Midwife explained that:

*“There are no gender gaps in mobile ownership among the Midwives but for pregnant women gender is a barrier as most mobile phones are owned by their husbands”*. *“Men can buy a mobile phone that women cannot afford to buy because men control the household income … gender gaps in mobile ownership is not a gender discrimination issue, it is an income issue”.*

### 3.4 Lack of mobile network connectivity

Lack of network connectivity in rural areas emerged as one of the most prominent barriers for mhealth technology. It was highlighted that, mobile network connectivity has not gained nationwide coverage in the rural areas affecting the usage of mobile phones by these pregnant women. Even in some rural areas where there are mobile network connectivity, their services are erratic and does not support full scale mhealth service provision. Lack of mobile network connectivity was expressed by participants:

These were how the pregnant women expressed this:

*“… they are not able to get us all the time on phone …. some of our places you have to fine a special spot to stand before you can make calls, once you leave that spot nobody can get you through phone again”*

*“What the Nurses are doing is good but just that network in this area is not good at all, you people can tell the MTN people to come and work on it for us”.*

### 3.5 Lack of electricity in some rural areas

All respondents agreed that lack of electricity for charging phones was an issue, particularly in rural areas. One pregnant woman said:

*“Those who have the phones and those who do not are the same because there is no light to charge the phones. It can take weeks before someone can charge her phone after the battery has ran down”.*

Views expressed by Midwives:

*“Access to electricity is a challenge. Rural households and most of our health facilities don’t have access to electricity… if you are in rural areas, you may not charge your phone for over one week”.*

*“Those villages without electricity, the charging of the phone is a problem. They may have the phone, but they may not get the power to maintain the phone. That's one of the challenges that we have in these communities”.*

### 3.6 Low female literacy rate at household level

Low female literacy rate was seen by respondents as a major barrier for the adoption of an mhealth application targeting pregnant women with messages. Quotes from some pregnant women:

*“Some of us cannot even read simple text messages … this is a problem. We may not even know a message has come left alone to read it”*

*“They have been given us text messages, but some of us cannot read… the other issue is skills ….you know some of us don't have the know-how to use the phone”*

From a Midwife:

*“If you are targeting pregnant mothers you should provide health messages in audio formats in addition to the text messages because the literacy status of women is limited”.*

### 3.7 Cultural barriers

Cultural barriers were identified by respondents as an impeding factor that hinders the utilization of maternal services that mhealth sought to increase: These were the expressions from pregnant women:

*“… sometimes the elderly women discourage us from such messages…they will tell you, if you know you are faithful to your husband you can carry you pregnancy to term and deliver, nothing will happen to you ….and that, the Nurses are only preparing you to come and deliver at their place”.*

*“The Nurses always want you to do what they want, they don’t want to give small chance to also do our local things”.*

The Midwives also have these to say:

*“In most rural areas, pregnant women don’t give birth at health facilities because of cultural issues… they have misguided information regarding diseases and spirits... to them mhealth application messages are meant to entice them to deliver at a health facility and therefore will rather avoid anything that has to do with mhealth”.*

*“Pregnant women in this our area prefer to deliver at home to proof that they are faithful to their husbands ….they will therefore try to avoid these messages that are encouraging them to attend antenatal and deliver at the health facility”.*

## 4 Discussion

The study explored pregnant women's awareness on mhealth and the challenges they face in using mhealth technology to improve maternal health in rural Ghana's Upper West Region (UWR).

Respondents were aware of the mhealth intervention in their districts and majority of the respondents cited phone knowledge as one of the most important factors influencing mhealth adoption and use. According to the respondents, learning at least one basic phone skill opens up a lot of possibilities for mhealth adoption and use. This is confirmed by a study that knowledge of mhealth technology was critical in influencing technology adoption in health care [18]. The users' understanding of the system and the benefits that result from its use is defined as usefulness. Acceptability will be very low if users do not find the system useful [18]. The study discovered that awareness on mhealth influenced the use of mobile phones to provide maternal and child health services by primary health care providers. Our findings are consistent with other studies in that, awareness toward mobile health applications can influence adoption and use in low-resource settings for maternal and child health care [10].

Pregnant women with limited literacy skills are unable to obtain basic maternal health information. Mobile phones can help pregnant women improve their maternal health literacy and information capabilities, in addition to providing access to maternal health care. As can be seen, the user participants' low levels of education prevented them from using their mobile phones for healthcare. Majority of users are unable to use their mobile phones for healthcare due to their inability to read and write. For example, because they are unable to operate their phone options or comprehend the message, they rarely read text messages or act on them. This finding is consistent with findings of a study conducted in Ejisu-Juaben municipality in Ghana by Agyemang-Duah et al., 2019, which reported that most rural healthcare users did not use mHealth because half of them were unaware of how to use text messages [19]. Some studies have suggested that users be trained and actively participate in the system evaluation process to improve their understanding of the system. The ability to meet the needs of users is at the heart of a successful mHealth system [20].

The cost of owning a phone and using several of its features had a negative impact on mHealth's for maternal health services. As a result, many pregnant women are forced to share their cell phones with their families. Naturally, sharing a cell phone among family members is linked to a lack of or very low income [18], which is common in the Upper West Region where the study was conducted.

According to the findings, lack of electricity and poor telecommunication infrastructure are barriers to pregnant women using their mobile phones to access mHealth services. The majority of people without reliable electricity live in Sub-Saharan Africa, where approximately 6 out of 10 people lack access, and those who have access to the electrical grid may experience more frequent blackouts and brownouts (50–4600 h per year) due to capacity shortages and infrastructure failures, forcing the population to seek alternative energy sources, most commonly diesel generators [21]. A stable and accessible power supply is a key consideration for mhealth adoption by pregnant women with low battery life on their mobile devices [21].

In Africa a man believes that women are solely responsible for all household chores, implying that women must care for their families and households regardless of the circumstances. Indeed, the findings show that many pregnant women are so preoccupied with their household responsibilities that they forget to seek maternal health care.

### 4.1 Limitations of the study

The qualitative study is obviously limited by its size as more pregnant women and midwives could have also provided additional useful information. In addition, we did not compare the findings among the three study districts in the Region. Finally, the findings are limited to the study area and therefore cannot be generalized to other settings. However, the depth of the interviews yielded clear issues that are useful for the study area, Ghana and the research community. Thus, we believe that the findings reflect the prevailing situation among the respondents in the study site. This is most likely the first study to be conducted on mhealth intervention in the region. Thus, the findings are useful for the implementation of an mhealth programme and foundation for future research.

## 5 What this study adds

Respondents were aware of the mhealth intervention in their districts.

Phone knowledge was a determinant to mhealth adoption in the study settings.

Lack of electricity was a barrier to mhealth adoption in the study settings.

## 6 Implications for policy and practice

⁃ mhealth intervention has the potential to reduce cost and geographical barriers associated with accessing maternal and child health services in rural settings.
⁃ Health policy-makers and implementers continually face maternal and child health services problems. The findings of the study show that, a scale-up of mHealth intervention could offer a solution to these problems.
⁃ The findings of the research show that, mHealth strategies have the potential to improve maternal and child health service utilization but face certain challenges that need urgent attention.

## 7 Conclusion

The Savanna Signatures mhealth pilot intervention is widely known by pregnant women but some challenges exist that impeded the smooth implementation of the intervention. These findings are of clear public health importance and are relevant to policy-makers in this area of service delivery and use. Policy-makers and program implementers should be cautious of the challenges of scale-up. To achieve this will require adequate support from governments, stakeholders, policy-makers, and program implementers.

## Ethic approval and consent to participate

This study was reviewed and approved by the Institutional Review Board of Navrongo Health Research Centre (Ethical Approval ID: NHRCIRB272). Written informed consent was obtained from each respondent prior to participating.

## Funding

The study was funded by 10.13039/501100008627Global Affairs Canada through Savanna Signatures, an NGO in Northern Ghana. The manuscript represents the views of the authors and not those of Savanna Signatures or Global Affairs, Canada.

## Declaration of competing interest

The authors declare that they have no known competing financial interests or personal relationships that could have appeared to influence the work reported in this paper.
